# Supplementary material for: A non-conserved amino acid variant regulates differential signalling between human and mouse CD28
Source: Nat Commun. 2018 Mar 14;9:1080. doi: 10.1038/s41467-018-03385-8 (PMC5852078; doi:10.1038/s41467-018-03385-8)
Supplement: Supplementary file 1 — Supplementary Information [file 41467_2018_3385_MOESM1_ESM.pdf]

Supplementary information

A non-conserved amino acid variant regulates differential signalling between human and mouse CD28

Porciello et al.

Supplementary Figure 1
Supplementary Figure 2
Supplementary Figure 3
Supplementary Figure 4
Supplementary Figure 5
Supplementary Figure 6
Supplementary Figure 7
Supplementary Figure 8
Supplementary Figure 9
Supplementary Figure 10
Supplementary Figure 11
Supplementary Figure 12
Supplementary Figure 13
Supplementary Figure 14
Supplementary Figure 15

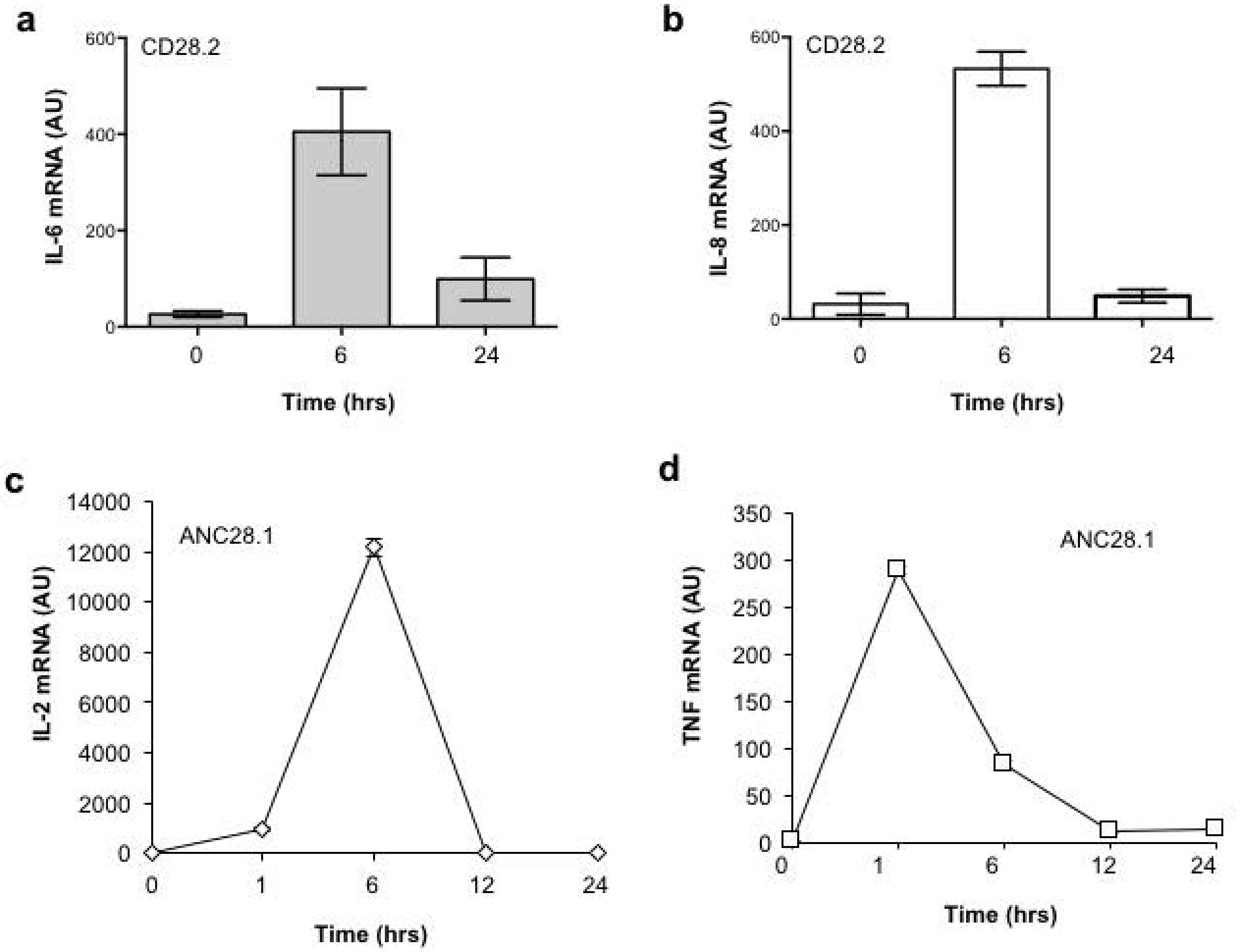

Supplementary Figure 1. Kinetic analysis of cytokine mRNA production upon agonistic or superagonistic CD28 stimulation of human $CD4^+$ T cells. (a-d) Human $CD4^+$ T cells were stimulated with 2 $\mu g\ ml^{-1}$ of crosslinked agonistic CD28.2 (**a, b**) or superagonistic ANC28.1 Abs (**c, d**) for different times and IL-6 (**a**), IL-8 (**b**), IL-2 (**c**) and TNF (**d**) mRNA levels were measured by real-time PCR. Data are expressed as arbitrary units (AU) normalized to GAPDH. Bars show the mean ± SD. (**a, b**) Data derive from three HD. (**c, d**) One experiment representative of three is shown. Statistical significance was calculated by Mann-Whitney test (**a, b**) or Student's t test (**c, d**). P values were < 0.05

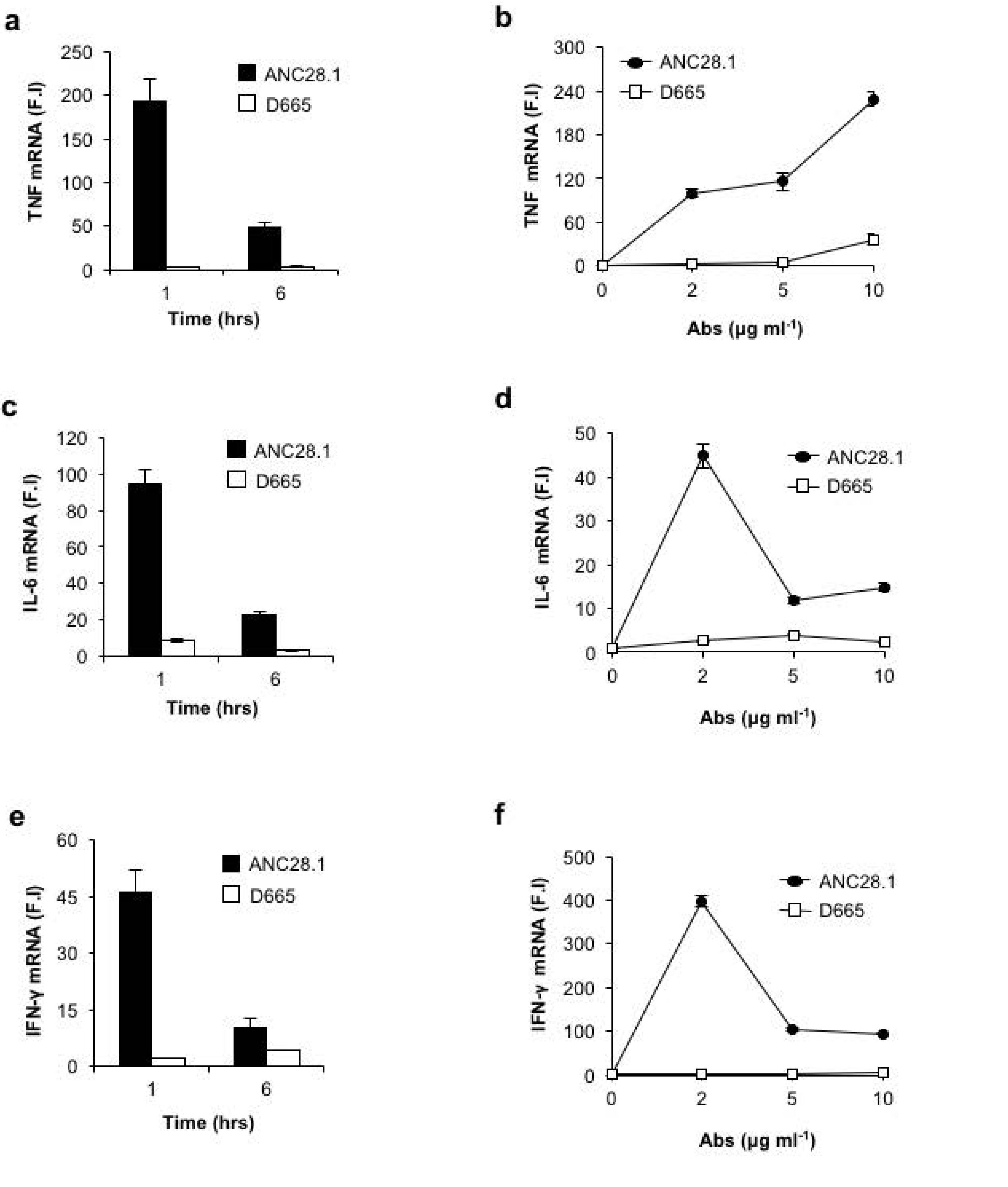

Supplementary Figure 2. Cytokine mRNA production upon superagonistic CD28 stimulation of human and mouse $CD4^+$ T cells. (a, c, e) Human or mouse $CD4^+$ T cells were stimulated with 2 μg ml^{-1} of crosslinked CD28SAbs (human ANC28.1 or mouse D665) for 1 h or 6 h and TNF (**a**), IL-6 (**c**), IFN-γ (**e**) mRNA levels were measured by real-time PCR. (**b, d, f**). Human or mouse CD4+ T cells were stimulated for 1 h with different concentrations of crosslinked CD28SAbs (human ANC28.1 or mouse D665) and TNF (**b**), IL-6 (**d**) and IFN-γ (**f**) mRNA levels were measured by real-time PCR. Data are expressed as fold inductions (F.I.) over the basal level of cells stimulated with control Ig. Bars show the mean ± SD of culture duplicates. One experiment representative of three is shown. Statistical significance was calculated by Student's t test. P values were < 0.05

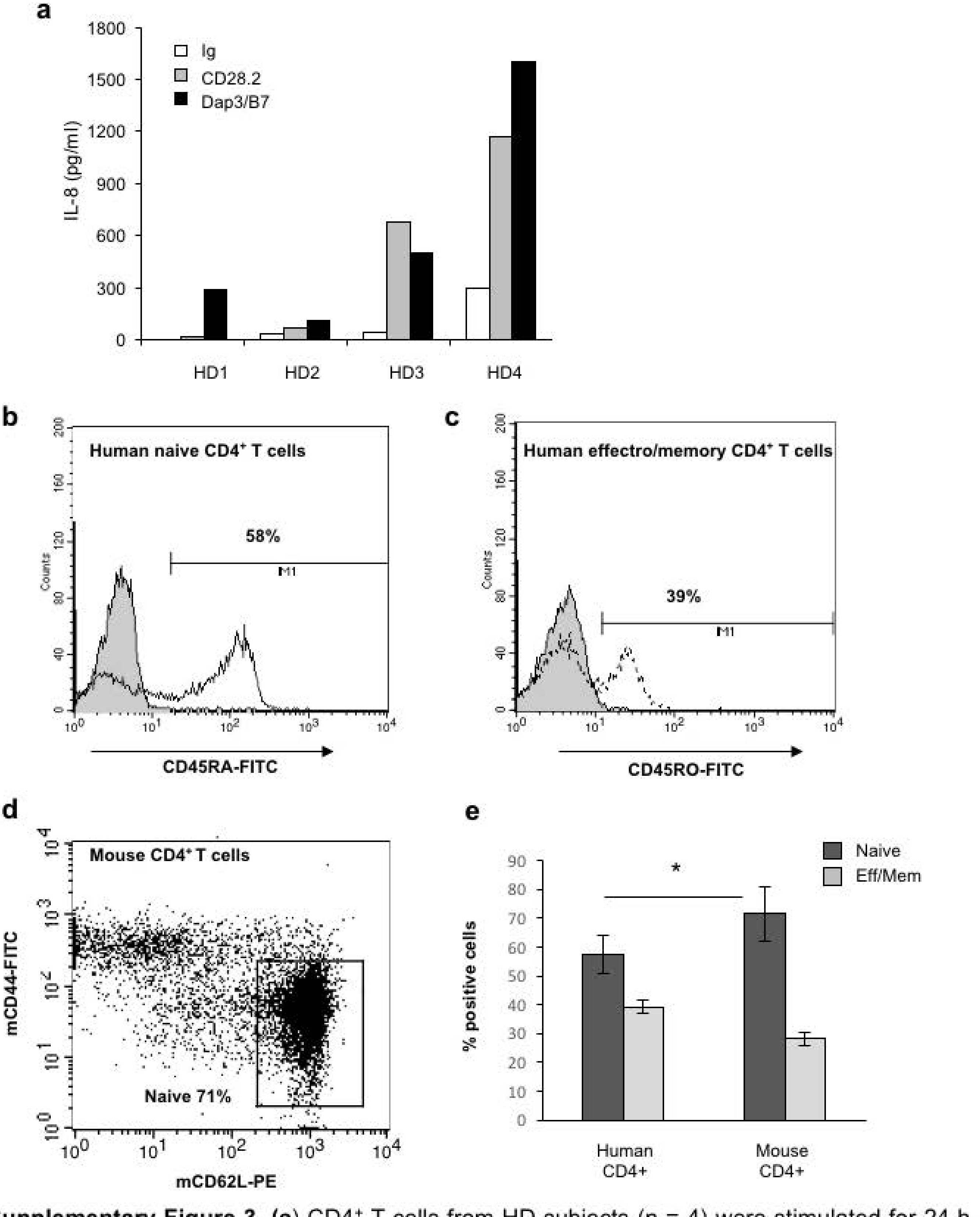

Supplementary Figure 3. (**a**) CD4$^+$ T cells from HD subjects (n = 4) were stimulated for 24 h with isotype control mAb (Ig) or 2 μg ml^{-1} crosslinked anti-CD28.2 Abs, or adherent Dap3/B7 cells. IL-8 levels in culture supernatant were measured by using BD CBA FLEX SET assays. (**b, c**) FACS analysis of CD45RA (**b**) and CD45RO expression of human peripheral blood CD4$^+$ T cells. (**d**) FACS analysis of CD44 expression of mouse splenic CD4$^+$ T cells. (**e**) The percentage of human naïve CD4+ T cells expressing CD45RA (hCD45RA) or effector/memory (Eff/Mem) CD45RO (hCD45RO) from HD (n = 3) and mouse naïve (CD44^{low}CD62L$^+$) or effector/memory (CD44$^+$CD62L$^{Low/-}$) CD4$^+$ T cells (n = 5) were calculated. The results express mean percentage of positive cells ± SD. (*) $P < 0.05$ calculated by Student's t test.

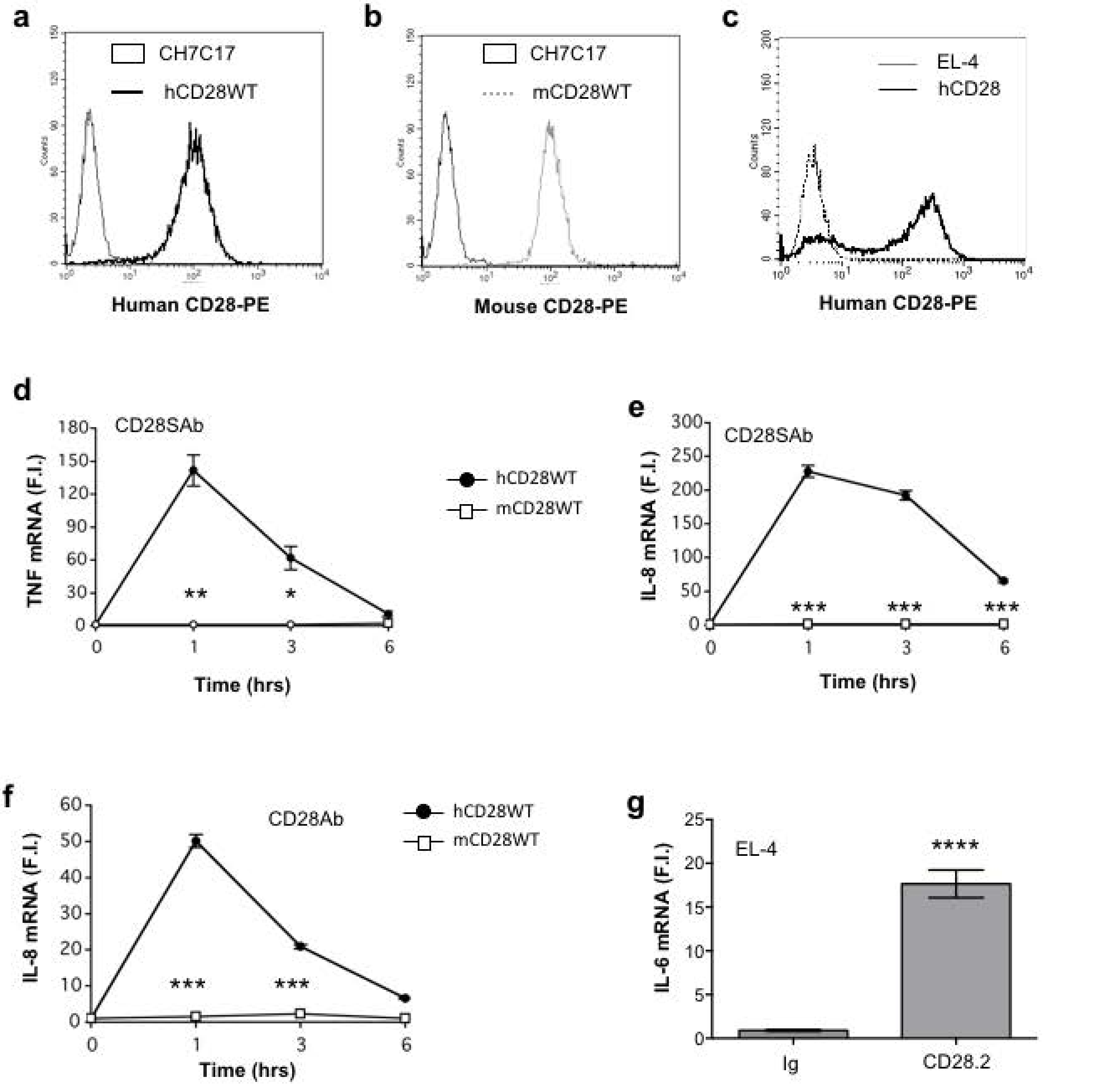

Supplementary Figure 4. Cytokine mRNA production upon superagonistic or agonistic CD28 stimulation of human CH7C17 Jurkat or mouse EL-4 cells expressing human or mouse CD28. (**a-c**) FACS analysis of CH7C17 Jurkat cells (**a,b**) or mouse EL-4 cells (**c**) expressing human CD28 (hCD28, **a, c**) or mouse CD28 (mCD28, **b**) with phycoerythrin-conjugated anti-CD28 Abs (CD28-PE). (**d-g**) Real-time PCR mRNA levels of the indicated cytokines in CH7C17 cells expressing hCD28WT or mCD28WT or mouse EL-4 cells expressing hCD28 (**g**), stimulated with control Abs (Ig) or crosslinked CD28SAb (human ANC28.1 or mouse D665) or CD28Ab (human CD28.2 or mouse 37.51) for the indicated times or 6 h (**g**). Bars show the mean fold inductions (F.I.) ± SD of one experiment representative of three. (*) P < 0.05, (**) P < 0.01, (***) P < 0.001, (****) P < 0.0001 calculated by Student's t test.

a

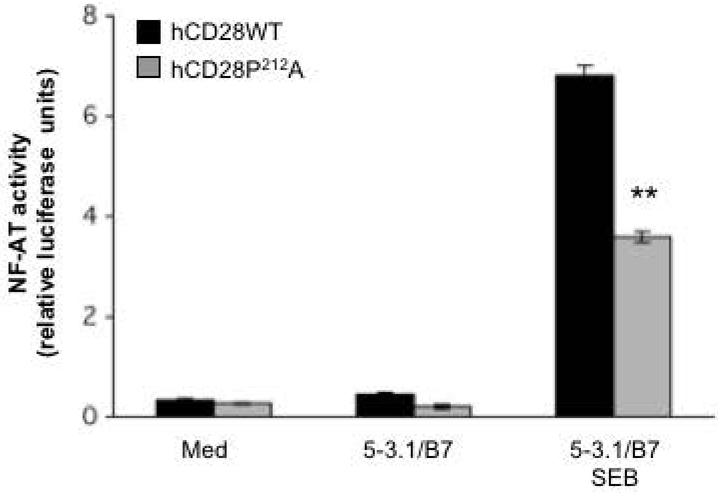

b

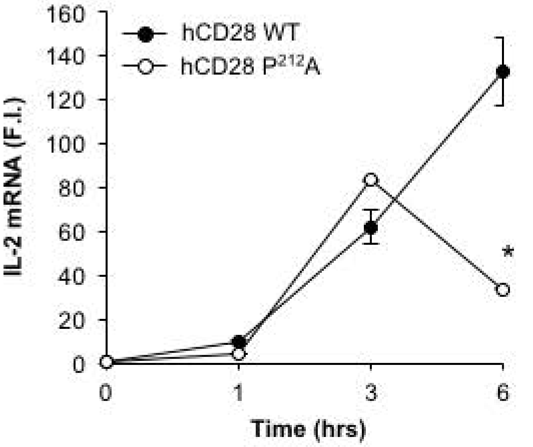

Supplementary Figure 5. P^{212}A substitution within the C-terminal proline rich motif ($YQPP^{212}$) of CD28 impairs NF-AT and IL-2 gene expression in response to TCR and CD28 co-engagement. (**a**) NF-AT luciferase activity of CH7C17 cells expressing hCD28WT, hCD28P^{212}A mutant stimulated with medium (Med) or 5-3.1/B7 cells pre-pulsed or not with 1 μg ml^{-1} SEB. The results are expressed as the mean of luciferase units ± SD after normalization to GFP expression. The data are representative of three independent experiments. (**b**) CH7C17 cells expressing CD28WT or hCD28P^{212}A mutant were stimulated with 2 mg ml^{-1} anti-CD3 (UCHT1) and CD28.2 Abs for different times. IL-2 mRNA levels were measured by real-time PCR and values, normalized to GAPDH, were expressed as fold inductions (F.I.) over the basal level of cells stimulated with isotype control Ig. Bars show the mean ± SD of one experiment representative of three. Statistical significance was calculated by Student *t* test. (*) $P < 0.05$, (**) $P < 0.01$

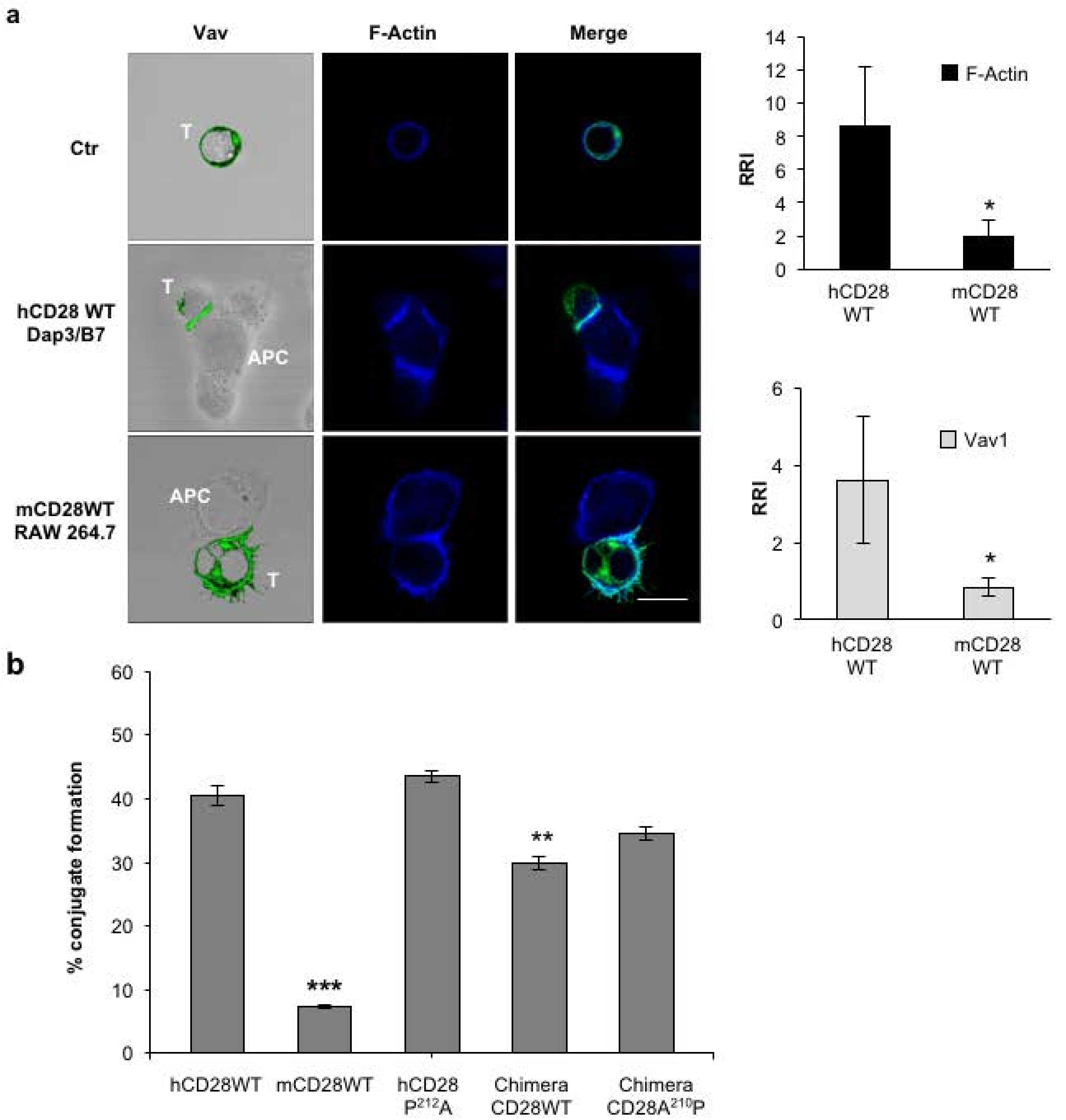

Supplementary Figure 6. (**a**) Confocal analysis of GFP-Vav1 (green) and F-actin (blue) recruitment in JCH7C17 cells expressing human (hCD28WT) or mouse (mCD28WT) CD28 stimulated in the absence (Ctr) or presence Dap3/B7 or RAW 264.7 cells. The scale bar represents 10 μm. Graphs represent the mean relative recruitment index (RRI) ± SD of fifteen conjugates analysed in each group (right panels). (**b**) hCD28WT, or mCD28WT, or hCD28P^{212}A mutant or chimera containing the extracellular and transmembrane domain of human CD28 and the cytoplasmic tail of mouse CD28 (Chimera CD28WT) mouse CD28 mutant (Chimera CD28A^{210}P) were stimulated for 5 min with Dap/B7 cells. Conjugate formation was measured by FACS and expressed as the mean percentage ± SD of three independent experiments performed in triplicates. (*) P < 0.05 (**) P < 0.01, (***) p < 0.001 calculated by Student's t test.

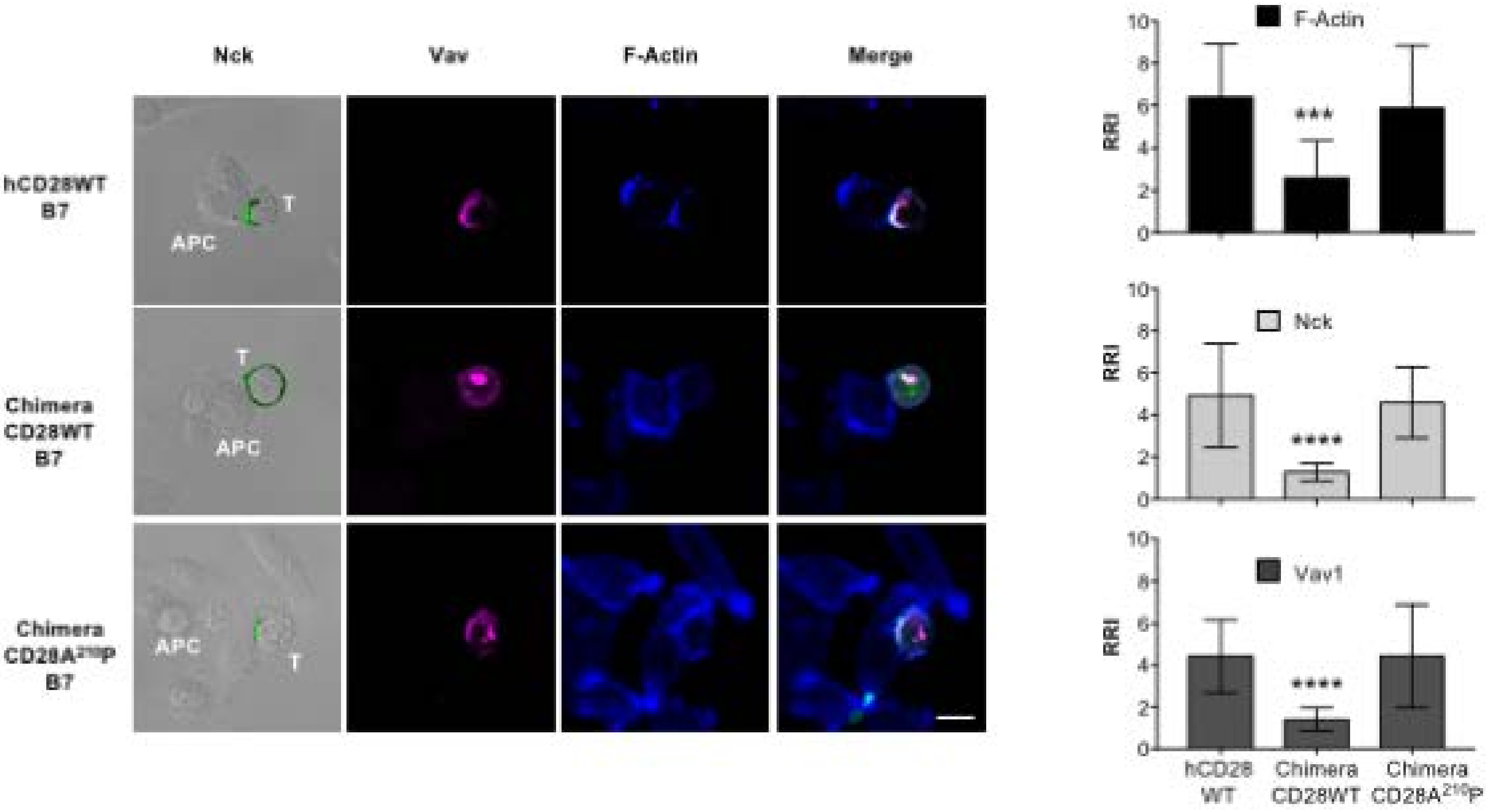

Supplementary Figure 7. Nck and Vav co-recruitment in Chimera CD28WT and Chimera A^{210}P. Human CD28 WT (hCD28) or chimera containing the extracellular and transmembrane domain of human CD28 and the cytoplasmic tail of mouse CD28 (Chimera CD28WT) or mouse CD28 mutant (Chimera CD28A^{210}P) were transfected with pm-Cherry Vav1 (magenta) vector together with GFP-Nck WT (green) and then stimulated for 15 min in the absence (Ctr) or presence of Dap/B7 cells (B7). After fixing and permeabilization F-actin was stained with 633-conjugated phalloidin (blue) and analysed by confocal microscopy. The scale bar represents 10 μm. The relative recruitment index (RRI) was calculated as described in Methods and represents the mean ± SD of fifteen conjugates analysed in each group. Mean values: Nck, hCD28WT = 4.9 ± 2.4, Chimera CD28WT = 1.3 ± 0.4, Chimera CD28A^{210}P = 4.4 ± 2.4; F-Actin, hCD28WT = 6.4 ± 2.5, Chimera CD28WT = 2.6 ± 1.7, Chimera CD28A^{210}P = 5.9 ± 2.9; Vav, hCD28WT = 4.4 ± 1.7, Chimera CD28WT = 1.4 ± 0.6, Chimera CD28A^{210}P = 4.4 ± 2.4. *** $P < 0.001$, **** $P < 0.0001$ calculated by Student's t test.

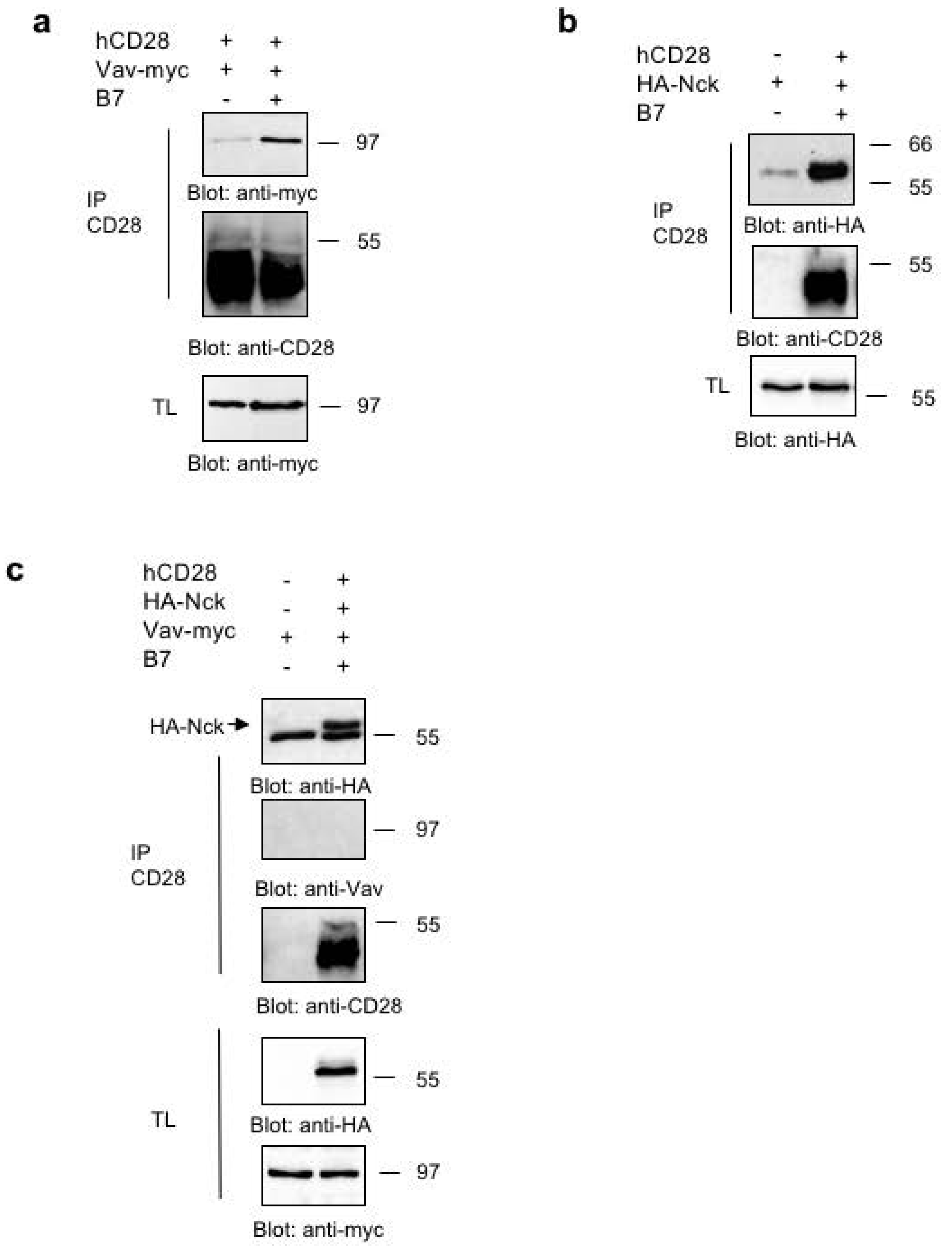

Supplementary Figure 8. **Association of human CD28 with Vav1 and Nck.** CH7C17 Jurkat cells were transfected with human CD28 WT together with myc-Vav1 (**a**) or HA-Nck (**b**) or Vav1-myc and HA-Nck (**c**), as indicated. Cells were then stimulated for 5 min with B7-negative (-) or Dap3/B7 cells (B7) and anti-CD28 immunoprecipitations (IP) were performed on cellular extracts. Anti-HA, anti-myc, anti-Vav and anti-CD28 western blottings were performed on anti-CD28 IP or TL. Data represent one of three independent experiments. The position of molecular weight markers, expressed in kDa, is indicated on the right

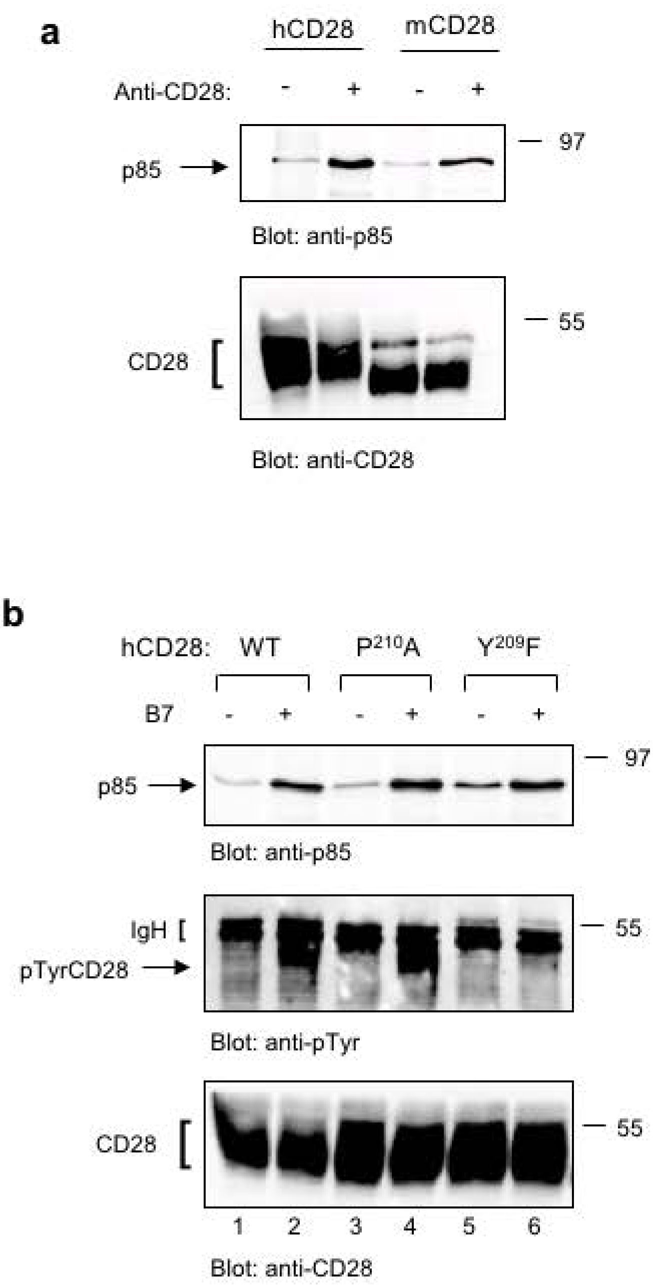

Supplementary Figure 9. (**a**) JCH7C17 cells expressing hCD28WT or mCD28 were stimulated for 5 min with agonistic CD28.2 or 37.51 Abs. Anti-p85 or anti-CD28 western btottings were performed on anti-CD28 immunoprecipitations (IP). (**b**) JCH7C17 cells expressing hCD28WT or CD28 hCD28P^{212}A or hCD28Y^{209}F mutants were stimulated for 5 min with adherent B7-negative cells (-) or Dap/B7 (B7) cells. Anti-p85 or anti-phosphotyrosine or anti-CD28 western btottings were performed on anti-CD28 IPs. The results are representative of three independent experiments. The position of molecular weight markers, expressed in kDa, is indicated on the right.

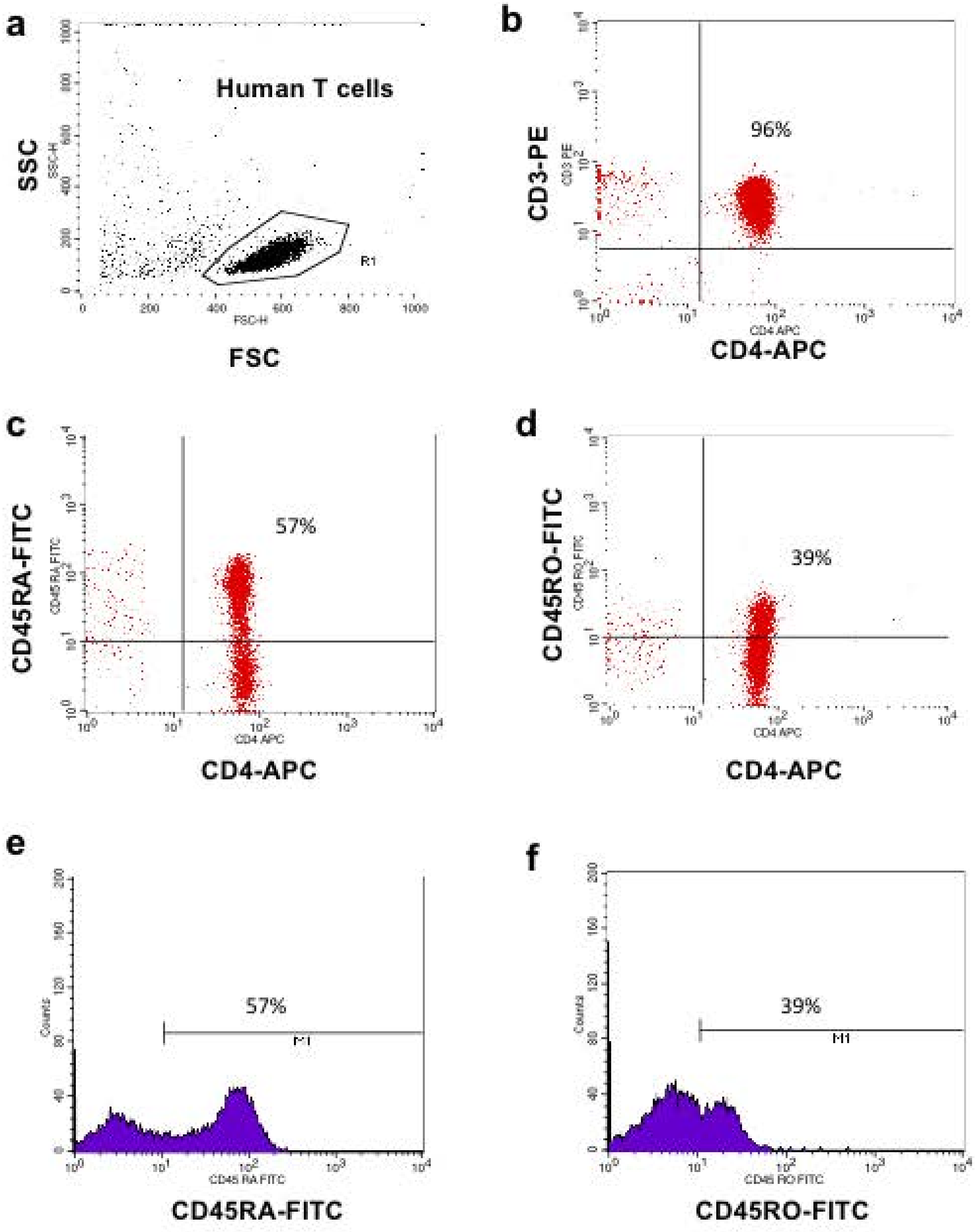

Supplementary Figure 10. Representative phenotypic analysis of human CD4⁺ T lymphocytes. Primary CD4⁺ T cells, identified by gating (R1) for SSC and FSC (**a**), were double stained with anti-CD3-PE plus anti-CD4-APC (**b**), or (anti-CD4-APC plus anti-CD45RA-FITC (**c**), or anrti-CD4-APC plus anti-CD45RO-FITC (**d**) Abs. (**e,f**) Histogram plots of $CD45RA^+$ (**e**) or $CD45RO^+$ (**f**) cells in gated $CD4^+$ T cells (**c,d**). The percentage of human $CD3^+CD4^+$ (**b**), or $CD4+CD45RA^+$ (**c,e**) or $CD4+CD45RO^+$ (**d,f**) T cells were then calculated.

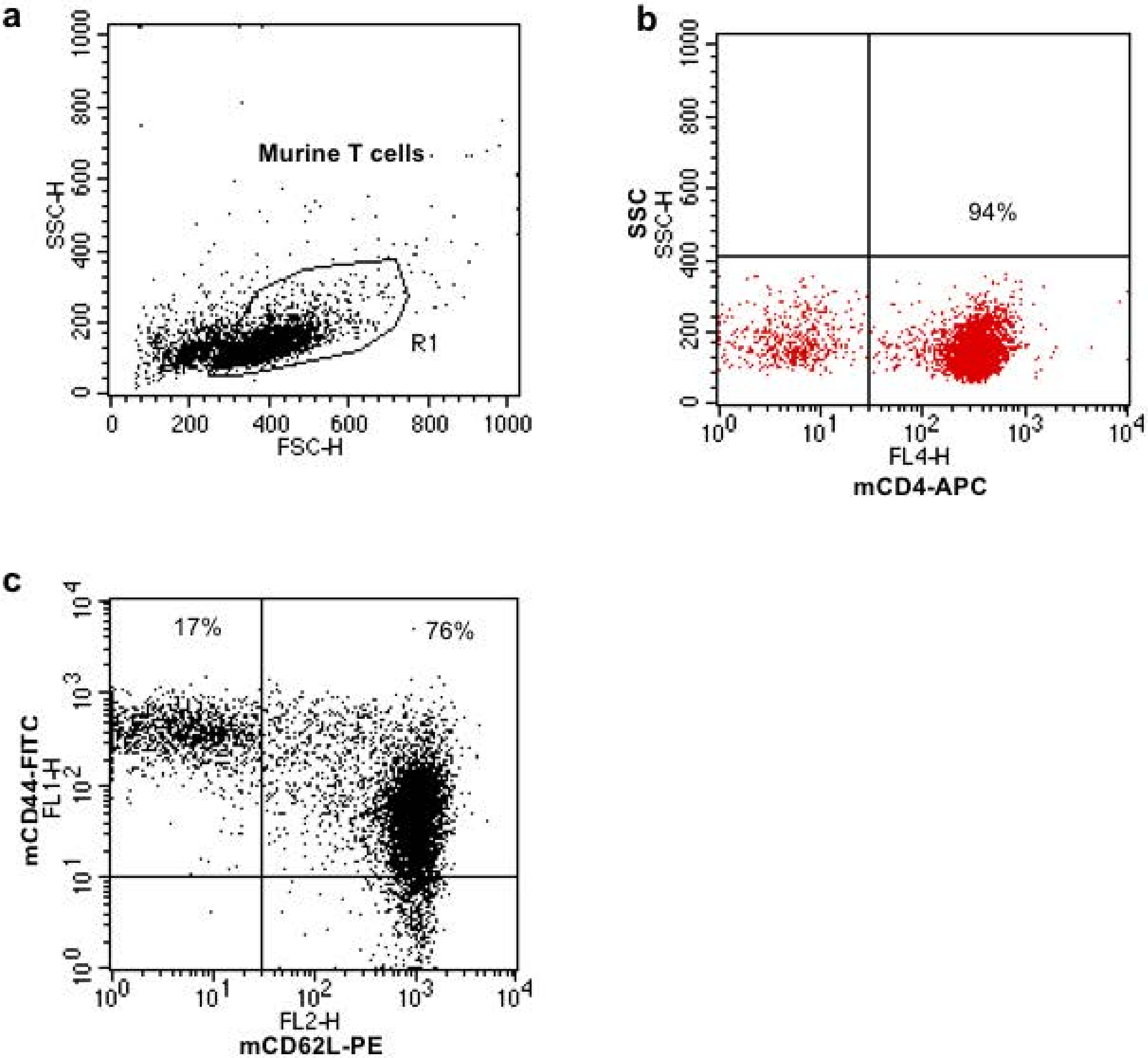

Supplementary Figure 11. Representative phenotypic analysis of murine $CD4^+$ T lymphocytes. Murine $CD4^+$ T cells, identified by gating (R1) for SSC and FSC (**a**) were stained with anti-CD4-APC plus anti-CD44-FITC and anti-CD62L-PE. Dot plots of $CD4^+$ vs SSC (**b**) or $CD62L^+$ vs $CD44^+$ cells in gated $CD4^+$ T cells (**c**) are shown. The percentage of murine $CD4^+$ (**b**), or $CD44^{low}$ $CD62L^+$ vs $CD44^+CD62L^{low/-}$ (**c**) T cells were calculated.

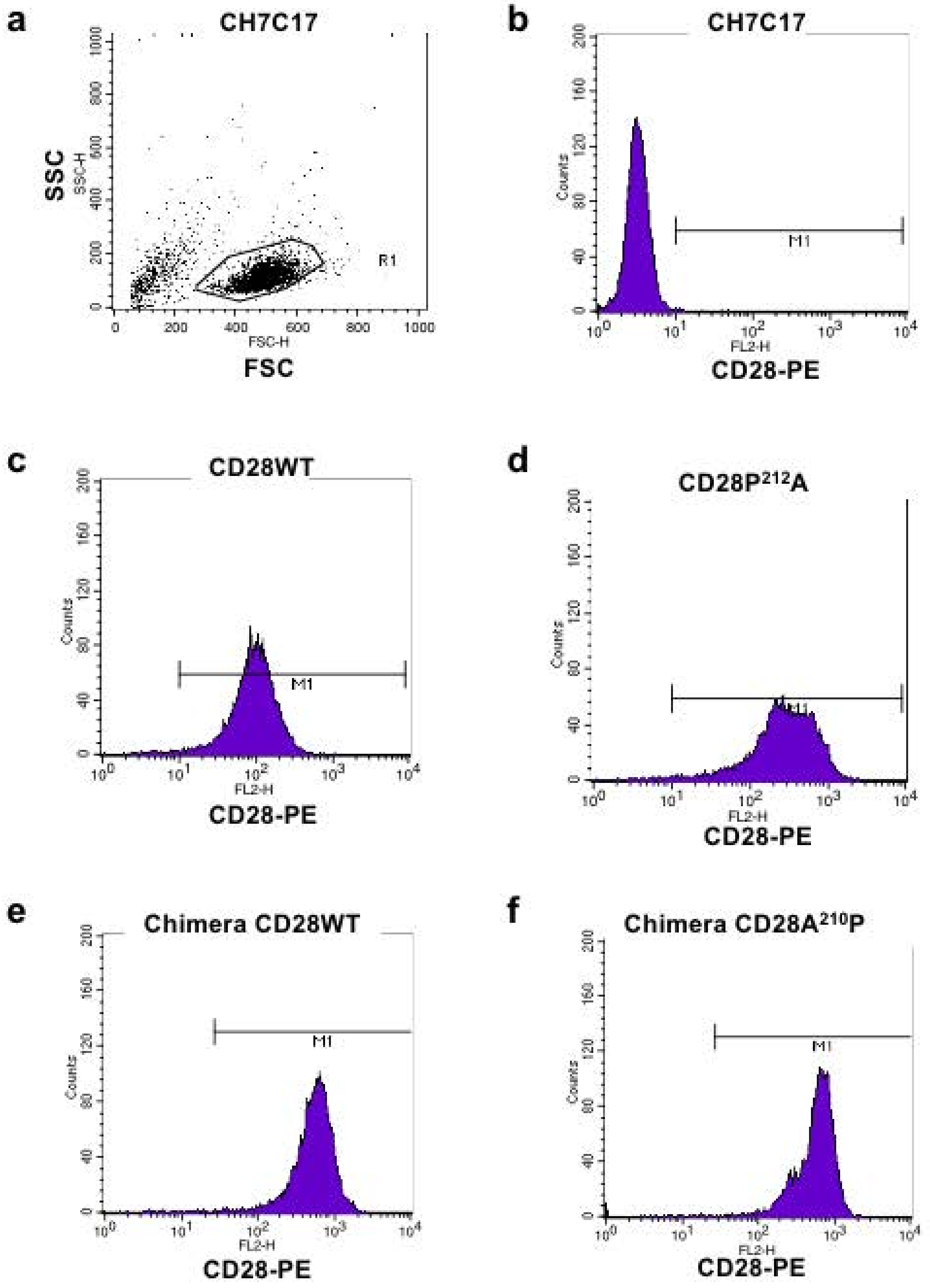

Supplementary Figure 12. Representative flow cytometry analysis of CD28 expression in CH7C17 Jurkat cells transfected with CD28 WT or mutants. CD28-negative CH7C17 Jurkat cells (**a,b**) or CH7C17 cells stable transfected with CD28WT (**c**), or CD28P^{212}A (**d**) or chimera CD28WT (e) or chimera CD28A^{210}P (**f**) mutants, were stained with anti-CD28-PE Abs and analysed by flow cytometry after gating (R1) for SSC and FSC. (**b-f**) Histogram plots of CD28-positive (M1) CH7C17 cells (**b**), or CD28WT (**c**), or CD28P^{212}A (**d**), or chimera CD28WT (**e**), or chimera CD28A^{210}P (**f**).

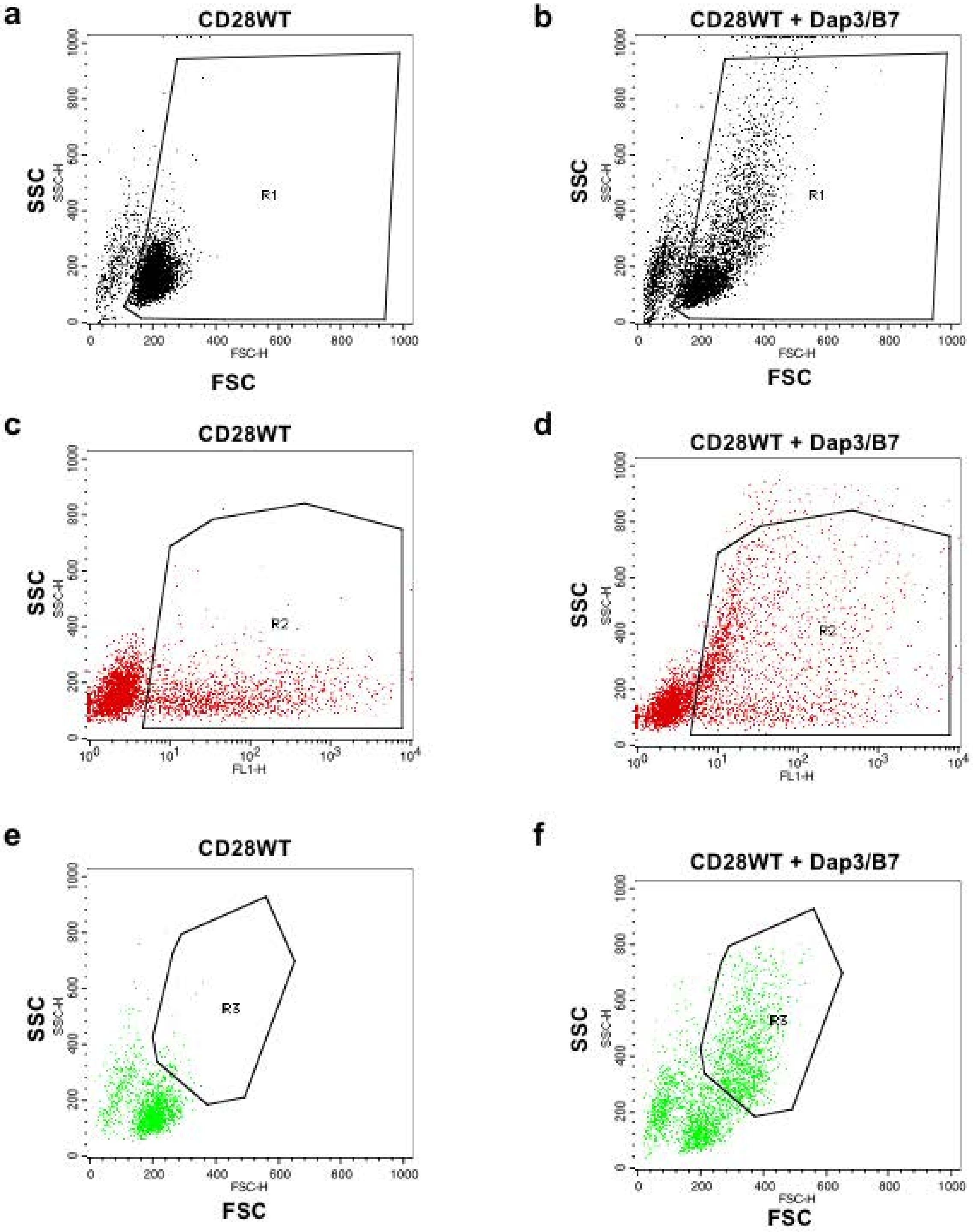

Supplementary Figure 13. Representative flow cytometry analysis of conjugates. CH7C17 Jurkat cells expressing human CD28WT were transfected with pEGFP construct, transfectants (3.5×10^6) were incubated for 5 min at 37°C in the absence (**a**,**c**,**e**) or presence (**b**,**d**,**f**) of Dap3/B7 cells (1.2×10^6) and analysed by FACS. (**a**,**b**) SSC vs FSC dot plots of CD28WT either unconjugated (a) or conjugated with Dap/B7 cells (**b**). R1 indicates the gate of alive cells. for Conjugates (**e**,**f**) were identified on GFP-positive cells (FL-1) within the R2 gate (**c**,**d**) by gating for SSC and FSC (R3).

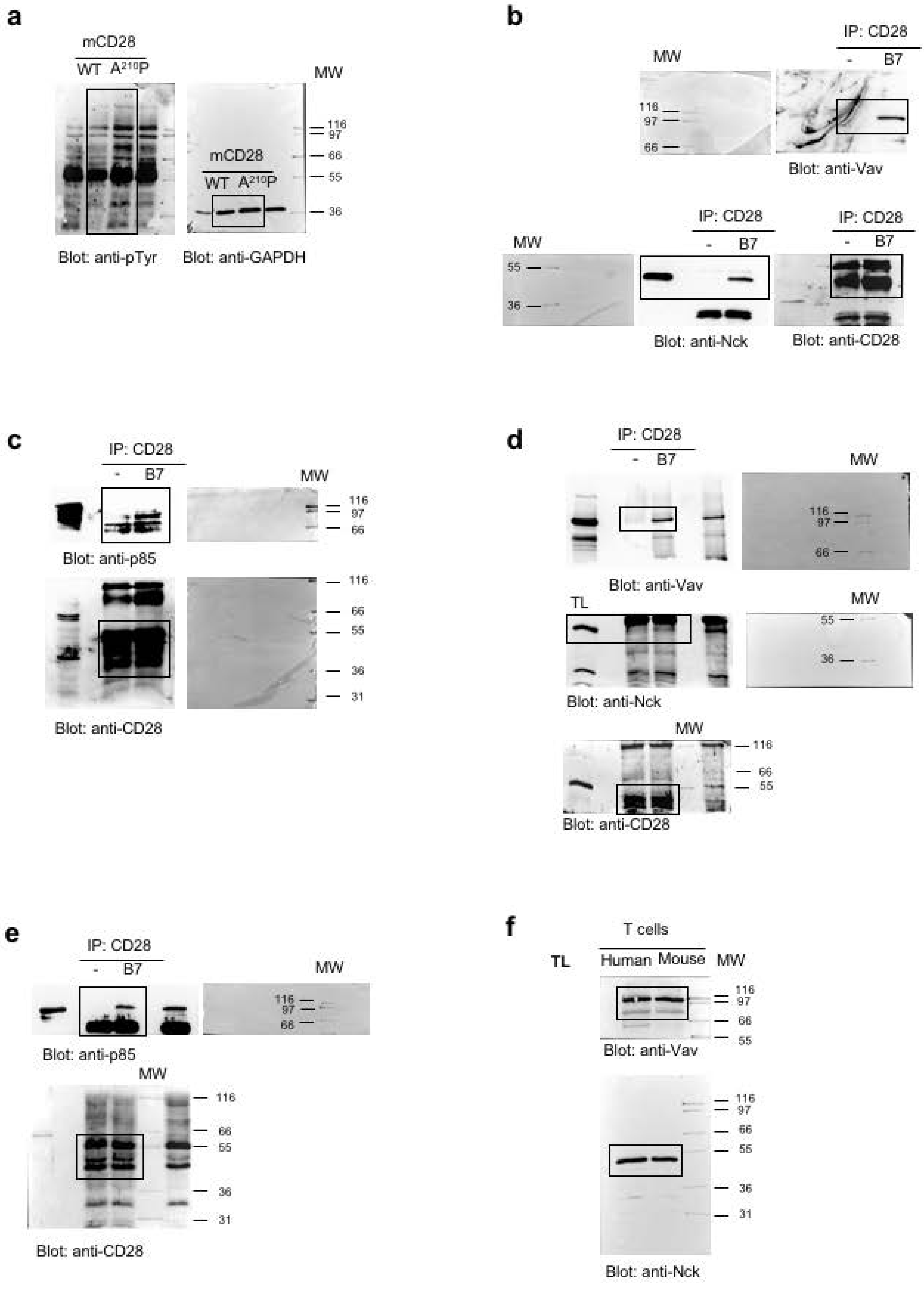

Supplementary Figure 14. Original data of immunoblots. (**a**) Immunoblot data corresponding to Fig. 4l. (**b**) Immunoblot data corresponding to Fig. 5a. (**c**) Immunoblot data corresponding to Fig. 5b. (**d**) Immunoblot data corresponding to Fig. 5c. (**e**) Immunoblot data corresponding to Fig. 5d. (**f**) Immunoblot data corresponding to Fig. 5e. MW = molecular weight markers in kDa.

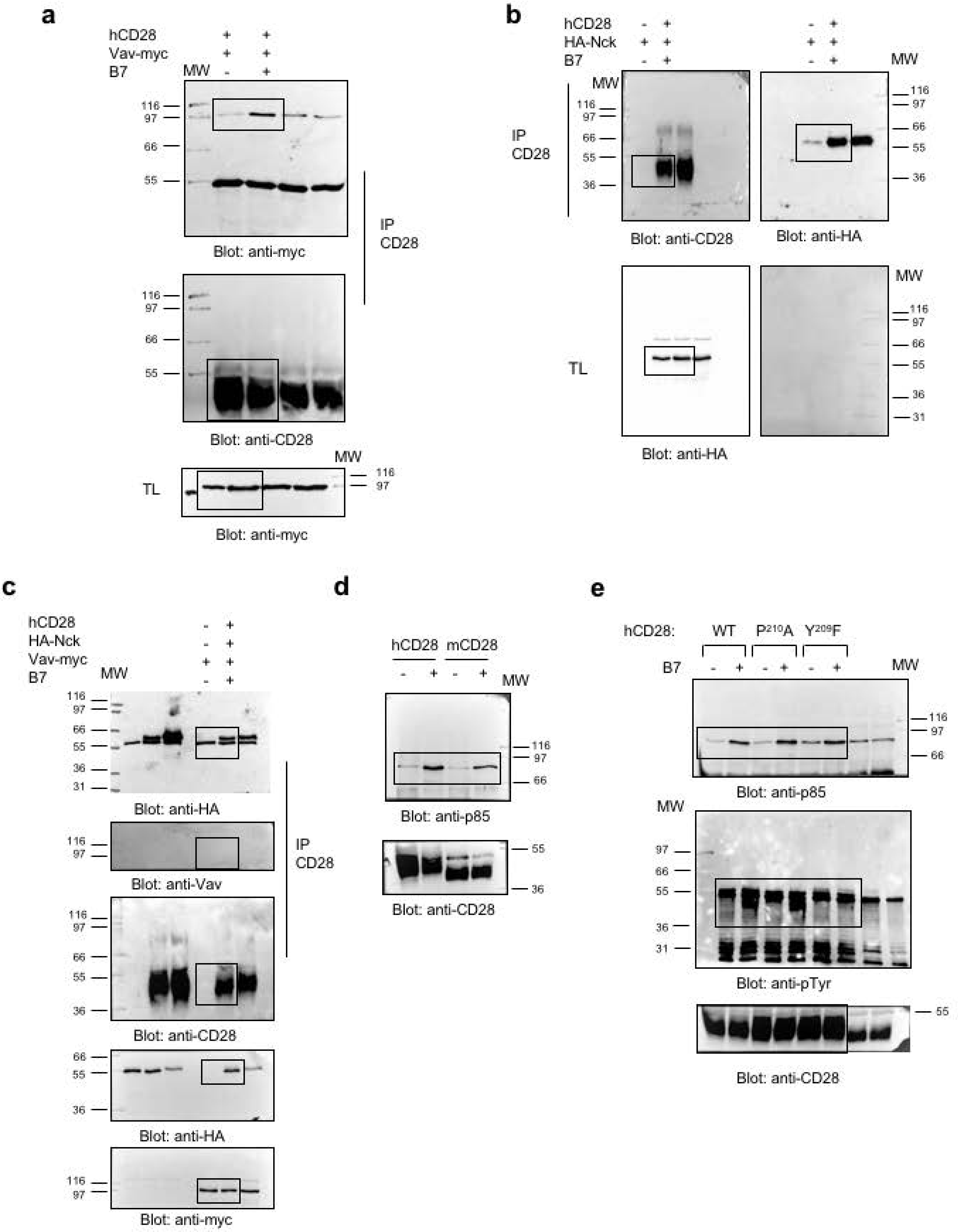

Supplementary Figure 15. Original data of immunoblots. (**a**) Immunoblot data corresponding to Supplementary Fig. 8a. (**b**) Immunoblot data corresponding to Supplementary Fig. 8b. (**c**) Immunoblot data corresponding to Supplementary Fig. 8c. (**d**) Immunoblot data corresponding to Supplementary Fig. 9a. (**e**) Immunoblot data corresponding to Supplementary Fig. 9b. MW = molecular weight markers in kDa.
